# Effects of Stimulus Frequency, Intensity, and Sex on the Autonomic Response to Transcutaneous Vagus Nerve Stimulation

**DOI:** 10.3390/brainsci12081038

**Published:** 2022-08-04

**Authors:** Hirotake Yokota, Mutsuaki Edama, Ryo Hirabayashi, Chie Sekine, Naofumi Otsuru, Kei Saito, Sho Kojima, Shota Miyaguchi, Hideaki Onishi

**Affiliations:** 1 Institute for Human Movement and Medical Sciences, Niigata University of Health and Welfare, Niigata 950-3198, Japan; 2 Department of Physical Therapy, Niigata University of Health and Welfare, Niigata 950-3198, Japan

**Keywords:** transcutaneous vagus nerve stimulation (tVNS), heart rate variability, stimulus frequency, stimulus intensity, sex differences, parasympathetic nervous activity

## Abstract

This study aimed to determine how transcutaneous vagus nerve stimulation (tVNS) alters autonomic nervous activity by comparing the effects of different tVNS frequencies and current intensities. We also investigated the sex-dependent autonomic response to tVNS. Thirty-five healthy adult participants were stimulated using a tVNS stimulator at the left cymba conchae while sitting on a reclining chair; tVNS-induced waveform changes were then recorded for different stimulus frequencies (Experiment 1: 3.0 mA at 100 Hz, 25 Hz, 10 Hz, 1 Hz, and 0 Hz (no stimulation)) and current intensities (Experiment 2: 100 Hz at 3.0 mA, 1.0 mA, 0.2 mA (below sensory threshold), and 0 mA (no stimulation)) using an electrocardiogram. Pulse widths were set at 250 µs in both experiment 1 and 2. Changes in heart rate (HR), root-mean-square of the difference between two successive R waves (RMSSD), and the ratio between low-frequency (LF) (0.04–0.15 Hz) and high-frequency (HF) (0.15–0.40 Hz) bands (LF/HF) in spectral analysis, which indicates sympathetic and parasympathetic activity, respectively, in heart rate variability (HRV), were recorded for analysis. Although stimulation at all frequencies significantly reduced HR (*p* = 0.001), stimulation at 100 Hz had the most pronounced effect (*p* = 0.001) in Experiment 1 and was revealed to be required to deliver at 3.0 mA in Experiment 2 (*p* = 0.003). Additionally, participants with higher baseline sympathetic activity experienced higher parasympathetic response during stimulation, and sex differences may exist in the autonomic responses by the application of tVNS. Therefore, our findings suggest that optimal autonomic changes induced by tVNS to the left cymba conchae vary depending on stimulating parameters and sex.

## 1. Introduction

The vagus nerve is known to innervate and significantly affect the function across different organ systems via the autonomic nervous system. Eighty percent of the vagus nerve is composed of afferent fibers, which play an important role in maintaining homeostasis by retrieving sensory input from organs controlled by the autonomic nervous system [1]. Afferent information from the vagus nerve enters the nucleus tractus solitarius (NTS) and then spreads through the locus coeruleus (LC) to the various cortical regions, such as the sensorimotor cortex, anterior cingulate cortex (ACC), and insular cortex (Ins) [2]. Transcutaneous Vagus Nerve Stimulation (tVNS) enables noninvasive electrical stimulation of vagal afferents and has been reported to improve symptoms of seizures, depression, migraine headaches, COVID-19, cardiac disease, and stroke [3,4,5,6,7,8,9,10]. Neurophysiological changes underpinning the effects of tVNS reveal that stimulation either to the tragus or cymba conchae, the auricular branches of the vagus nerve, changes heart rate (HR) and heart rate variability (HRV) [11,12]. Additionally, studies using functional magnetic resonance imaging (fMRI) have reported that tVNS increases activity, not only in the NTS and LC but also in projected cortical areas [13,14].

Despite recent findings on the effects of tVNS, an optimal stimulation method has not been standardized because of inconsistent application of tVNS parameters. Some reports indicate a decrease in HR and an increase in parasympathetic activity, immediately after tVNS, with a rebound in HR following the stimulation [11,12,15,16,17]. However, contrasting reports found no significant differences in using tVNS compared to sham stimulation of the earlobe [18]. Inconsistent stimulation criteria may have significantly impacted these studies and led to the contrasting results. Badran et al. examined the change in HR by tVNS to tragus with different stimulus frequency and pulse width combinations and reported a significant reduction in HR observed at 10 Hz or 25 Hz with 500 µs pulse width, with higher energy density conditions used in their experiment [15]. A meta-analysis on the effects of vagus nerve stimulation (VNS) on epilepsy patients also suggested that higher stimulus frequencies (in the range 1–30 Hz) were more effective for seizure suppression [19] and the effect remained consistent with a proportional increase in stimulus frequency. Although these studies examined the relatively low frequency band below 30 Hz, a study using fMRI to measure brain activity during tVNS demonstrated significantly higher activity in the NTS with 100 Hz stimulation than with low-frequency stimulation below 25 Hz [20]. Similarly, a study in rats found that stimulation ≥80 Hz was significantly more effective than stimulation ≤30 Hz in suppressing epileptic seizures [21]. Therefore, the possibility that tVNS in the high-frequency band may have a more pronounced effect on HR and autonomic nervous system activity warrants closer examination.

Changes in autonomic nervous system activity have also been observed at both low- and high-stimulation intensities [18]. In an animal study, high-intensity VNS delayed atrial conduction speed and induced atrial fibrillation [7]. Interestingly, low-intensity VNS has been reported to be effective in suppressing atrial fibrillation [10,22,23,24]. Furthermore, previous studies using implanted VNSs have reported a greater reduction in HR in proportion to current intensity [25]. However, the comparability of HR reduction using different tVNS current intensities to the left cymba concha versus implanted VNS and the overall impact on autonomic nervous activity is unknown. Furthermore, previous studies have shown that the response of HR and HRV to vagal stimulation is not always proportional and some studies claim that increasing the intensity of stimulation does not affect autonomic activity [18,26]. Additionally, it has been reported that sex differences in changes in autonomic activity were observed for the same stimuli [16]. These findings suggest that there may be sex-dependent differences in ideal stimulus intensities for changes in HR and HRV with tVNS. Moreover, stimulus-intensity-dependent changes need further evaluation to determine safe and therapeutically viable use of tVNS.

This study evaluated the tVNS-induced modulation of HR and HRV by comparing the frequency and stimulation-intensity-dependent effects of tVNS. We hypothesized that the effects of tVNS in the high-frequency band and at high intensities on HR would be more pronounced and that there is an ideal range of these parameters to reliably induce parasympathetic activity.

## 2. Materials and Methods

### 2.1. Participants

Thirty-five healthy adults (mean age: 21.2 ± 1.3 years) with no history or family history of epileptic seizures, no neurological, psychiatric, or cardiac diseases, and no history of external ear trauma participated in this study. Additional inclusion criteria were as follows: no metal implants in the body, no pregnancy, no alcohol or illicit drug dependence, and no daily medication. Twenty subjects participated in Experiment 1 and eighteen in Experiment 2, with three subjects participating in both Experiments 1 and 2. All study activities were conducted in accordance with the Declaration of Helsinki. This study was approved by the Ethics Committee of Niigata University of Health and Welfare, and all subjects were given sufficient explanation and provided their written informed consent.

### 2.2. Transcutaneous Vagus Nerve Stimulation (tVNS)

A battery-driven stimulation device (NEMOS, Cerbmed, Germany) was used for tVNS in Experiments 1 and 2. After wiping with alcohol-soaked cotton, two hemispheric titanium-stimulating electrodes were fitted to the left cymba concha. Saline-soaked sponges were attached to the electrodes to ensure optimal conductivity. The cymba conchae were used as a stimulation cite because they are innervated only by vagal afferent fibers and have reported clear activity in the NTS and LC by tVNS [13,27,28]. A comparison was made between the effects of the control condition, in which electrodes were worn but no stimulation was added, and the effects of the other conditions.

### 2.3. Experimental Procedures

**Figure 1:**
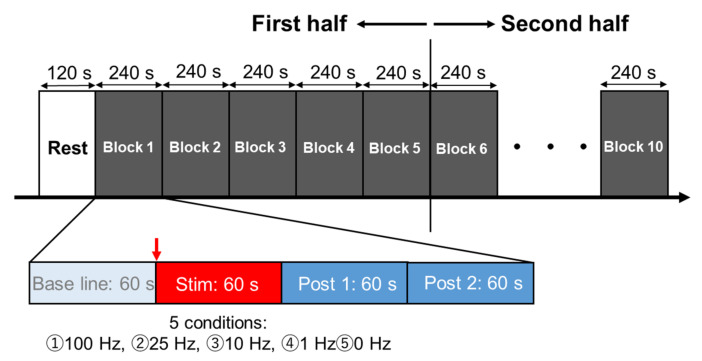
Experimental protocol in Experiment 1. Five stimulus frequencies (100 Hz, 25 Hz, 10 Hz, 1 Hz, 0 Hz) were randomly assigned to 5 blocks in the first half and 5 blocks in the second half.

**Figure 2:**
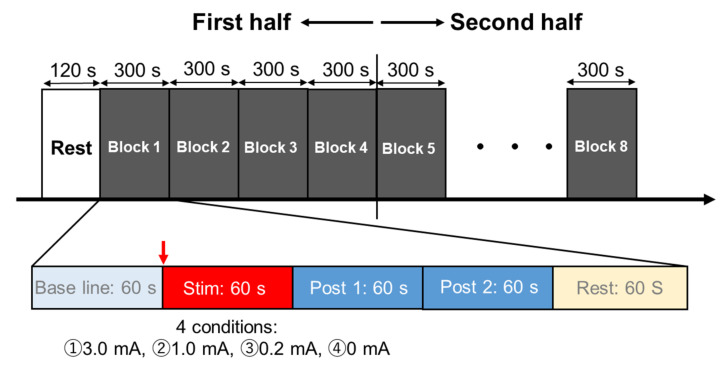
Experimental protocol in Experiment 2. The first half was divided into 4 blocks and the second half into 4 blocks, with four stimulus intensities (3.0 mA, 1.0 mA, 0.2 mA, 0 mA) assigned randomly to each block.

In both Experiments 1 and 2, the participants were seated in resting position on a reclining chair with armrests and the experiment was conducted between 9:00 a.m. and 12:00 p.m. in a quiet, dimly lit room with a room temperature of 22 °C to 24 °C. The participants were told to avoid alcohol and caffeine consumption 12 h before the experiment and to have breakfast at least 2 h before the experiment session. Each participant entered the laboratory after emptying his/her bladder, was fitted with a tVNS device and an electrocardiograph (ECG), and then rested in the chair used in this experiment for 20 min to calm autonomic nervous system activity. In Experiment 1, two sets of five stimulation frequencies; 100 Hz, 25 Hz, 10 Hz, 1 Hz, and 0 Hz (no stimulation) were randomly assigned to 10 blocks of 5 blocks each in the first and second halves, with each block containing a 1 min baseline, stimulation, Post 1 (1 min from immediately after termination of the stimulation), and Post 2 (1 min after Post 1) analysis windows where R-R intervals were counted (Figure 1). The current intensity was set at 3.0 mA, the maximum possible current for the device. In Experiment 2, all subjects were first measured for sensory thresholds using the staircase method from 0.1 mA in 0.1 mA increments until a participant reported sensation with a stimulus frequency of 100 Hz, the frequency that produced the most pronounced stimulation effect in Experiment 1. Next, 2 sets of 4 stimulus intensities were randomly assigned to 8 blocks each in the first and second halves. An intensity of 0.2 mA corresponded to below sensory threshold, 1.0 mA and 3.0 mA were above sensory threshold, and 0 mA (no stimulation) represented the control condition. In each block, R-R intervals were plotted every 1 min each in Baseline, during stimulation, Post 1, and Post 2 as in Experiment 1 (Figure 2). In Experiment 1, the subjective discomfort level to tVNS was measured using the Numerical Rating Scales (NRS) 10 s after the termination of the stimulation. In addition, a heartbeat rebound was observed immediately after stimulation. To clarify whether this rebound was a physiological response due to post-stimulus homeostasis or an effect of verbal NRS measurement, in Experiment 2, an additional 1 min pause was added between Post 2 and the baseline of following blocks, and the NRS was taken 10 s after the end of Post 2 during the pause.

### 2.4. Electrocardiogram

Two types of electrocardiography (ECG) devices, Bio Amp (AD Instruments , Colorado Springs, CO, USA) and LRR-05 (GMS Co., Ltd., Tokyo, Japan), were used to measure electrocardiograms because of their different analysis characteristics. Three Ag/AgCl surface electrodes (Blue Sensor, METS, Tokyo, Japan) were placed on the left and right clavicles and costal margins to record HR. The ECG signals were amplified by an amplifier (A-DL-720·140, 4 ASSIST), converted to A/D, and then stored on a personal computer using Power lab (AD Instruments, Colorado Springs, CO, USA) for offline analysis with a sampling frequency of 1 kHz and a bandpass filter from 0.5 to 35 Hz. Offline analysis was conducted with a Labchart 8 (AD Instruments, Colorado Springs, CO, USA) and MemCalc/Bonaly Light (GMS Co., Ltd., Tokyo, Japan). Real-time HR was monitored to ensure safety and displayed on a monitor, which was placed out of sight of the participant to avoid influencing their autonomic nervous activity by visual information.

### 2.5. Data and Statistical Analyses

Labchart8 (AD Instruments) was used to calculate HR and the root-mean-square of successive differences (RMSSD), which indicates the time-series change in the square root of the standard deviation of the interval between two consecutive R waves (R-R interval). The frequency components of each R wave were analyzed by the maximum entropy method using MemCalc/Bonary Light (GMS Corporation). HR and HRV data were averaged for each of the above 1 min analysis windows to compare the effects of tVNS at different frequencies (Experiment 1) and current intensities (Experiment 2). In addition, to examine the onset and duration of the stimulation effect in detail, we compared the HR change every 5 s for each condition. Power spectral analysis was performed by calculating area under the low-frequency (LF) (0.04–0.15 Hz) band, a component indicating sympathetic activity including parasympathetic activity, and high-frequency (HF) (0.15–0.40 Hz) band, a component indicating parasympathetic activity. LF/HF was then calculated to determine the balance of the autonomic activity. The higher the LF/HF value, the more sympathetic activity is dominant, and the lower the value, the more parasympathetic activity is dominant.

To evaluate the effects of tVNS on HR, RMSSD, and LF/HF, statistical analyses were performed using statistical software (SPSS; IBM). Normal distribution was observed for HR and RMSSD, and repeated measures two-way analysis of variance (ANOVA) with post hoc Bonferroni correction was performed for frequency and time factors in Experiment 1 and for stimulus intensity and time factors in Experiment 2 on the means of each autonomic index at baseline, during stimulation, Post 1 at 1 min after stimulation, and Post 2 at 1 min after Post 1. Since LF/HF was not normally distributed in both Experiment 1 and 2, Friedman tests were performed. To examine the onset and duration of the stimulus effect in detail, a repeated measures two-way ANOVA was performed after a normality test on the 5 s HR data, followed by Dunnett’s method as a post hoc analysis. Furthermore, Spearman’s rank correlation coefficient was used to test the relationship between baseline autonomic nervous activity and its change in stimulation period. The significance level 5% was considered as statistically significant in all tests.

## 3. Results

### 3.1. Effects of Different Stimulus Frequencies of tVNS on HR and HRV

**Figure 3:**
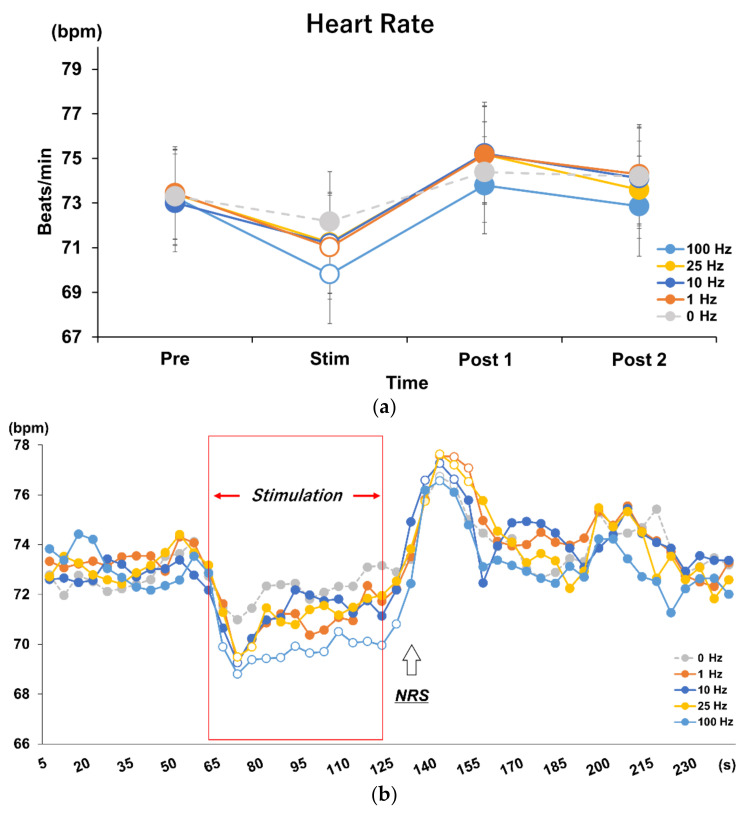
(**a**) Effects of different stimulus frequencies of tVNS on HR. The results of repeated measures two-way ANOVA showed main effects and interactions for frequency and time factors (F = 2.607, *p* = 0.003). The 100 Hz condition was the most effective in decreasing HR during stimulation compared to the other frequencies (*p* = 0.001). White dots indicate significant differences. (**b**) Time course analysis on every 5 s in each stimulus frequency condition. Post hoc analysis using Dunnett’s method revealed different effects during stimulation and rebound after stimulation for each frequency (*p* < 0.05). White dots indicate significant differences. Upward-pointing arrow indicates the timing of administration of the Numerical Rating Scale (NRS) for discomfort, which was taken 10 s after termination of the stimulation.

For Experiment 1, tVNS-associated changes in HR were normally distributed and repeated measures two-way ANOVA revealed a significant difference in the interaction between frequency and time (F = 2.607, *p* = 0.003). Post hoc analysis identified a significant decrease in HR during stimulation compared to baseline, Post 1, and Post 2, at all frequencies except the 0 Hz control condition (*p* = 0.001). Furthermore, the decrease in HR during stimulation was significantly greater in the 100 Hz condition compared to all other frequencies (*p* = 0.001) (Figure 3a).

### 3.2. Time Course Analysis on Every 5 s in Each Frequency Condition

Dunnett’s method was used to test the HR change every 5 s during each 240 s block (beginning of baseline measurement to the end of Post 2). At all stimulus frequencies, except the control condition, HR decreased immediately after the stimulus, reaching its lowest value after 10 s; then, HR gradually returned to baseline by the end of the stimulus, followed by a rebound after the end of the stimulus. At 100 Hz tVNS, the HR was always significantly lower than the baseline during the stimulus and up to 5 s after the end of the stimulus (*p* < 0.007). The post-stimulus rebound was significantly higher than baseline at 35 to 40 s after the end of stimulation (*p* = 0.023) and then returned to baseline. In the 25 Hz condition, HR decreased significantly from 10 to 20 s after stimulation (*p* < 0.025) and rebounded from 15 to 30 s after the end of stimulation (*p* < 0.01). At 10 Hz, HR decreased significantly only at 5 to 10 s after the onset of stimulation (*p* = 0.007) and rebounded from 10 to 25 s after the end of stimulation (*p* < 0.0004). At 1 Hz, HR decreased from 5 to 15 s after the onset of stimulation and rebounded from 15 to 30 s after the end of stimulation (*p* < 0.002). Only in 0 Hz, there was no decrease in HR during stimulation, but there was an increase in HR from 10 s to 25 s after the end of stimulation (*p* < 0.04) (Figure 3b).

### 3.3. Effects of Different Stimulus Intensities of tVNS on HR and HRV

**Figure 4:**
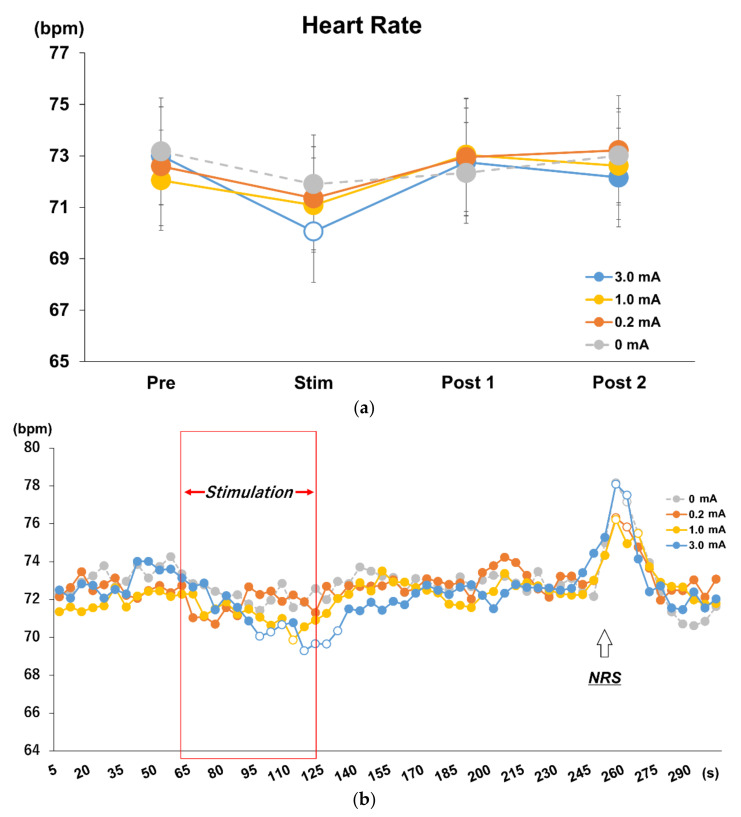
(**a**) Effects of different stimulus intensities of tVNS on HR. Repeated measures two-way ANOVA revealed main effects and interactions for intensity and time factors (F = 1.951, *p* = 0.049). Post hoc analysis revealed a decrease in HR during stimulation only in the 3.0 mA condition compared to baseline (*p* = 0.005), Post 1 (*p* = 0.003), and Post 2 (*p* = 0.023). (**b**) Time course analysis on every 5 s in each stimulus intensity condition. Post hoc analysis using Dunnett’s method revealed different effects during stimulation for each stimulus current intensity, and rebound was observed during NRS administration 10 s after the end of Post 2 (shown as an upward-pointing arrow) rather than at the end of stimulation (*p* < 0.05). White dots indicate significant differences.

In Experiment 2, as in Experiment 1, normality was found in the change in HR with tVNS, and a repeated measures two-way ANOVA revealed an interaction between stimulus intensity and time (F = 1.951, *p* = 0.049). Post hoc analysis revealed that only the 3.0 mA condition showed a significant decrease in HR during stimulation compared to Baseline (*p* = 0.005), Post 1 (*p* = 0.003), and Post 2 (*p* = 0.023). Significant HR differences were found during stimulation only with Post 1 in the 1.0 mA (*p* = 0.014) and 0.2 mA (*p* = 0.013) conditions, respectively (Figure 4a).

### 3.4. Time Course Analysis on Every 5 s in Each Stimulus Intensity Condition

Significant HR differences were found during stimulation only in Post 1 in the 1.0 mA (*p* = 0.014) and 0.2 mA (*p* = 0.013) conditions, respectively (Figure 4a). The significant rebound in HR immediately after stimulation observed in Experiment 1 was suspected to be due to the verbal response of NRS acquisition. Therefore, in Experiment 2, an NRS test was performed during the rest period after Post 2 and no rebound was observed immediately after stimulation, and a rebound similar to Experiment 1 was observed when an NRS test was performed in rest period after Post 2 (Figure 4b).

### 3.5. Correlation between Baseline and Stimulation Period in Experiment 1 and 2

**Figure 5:**
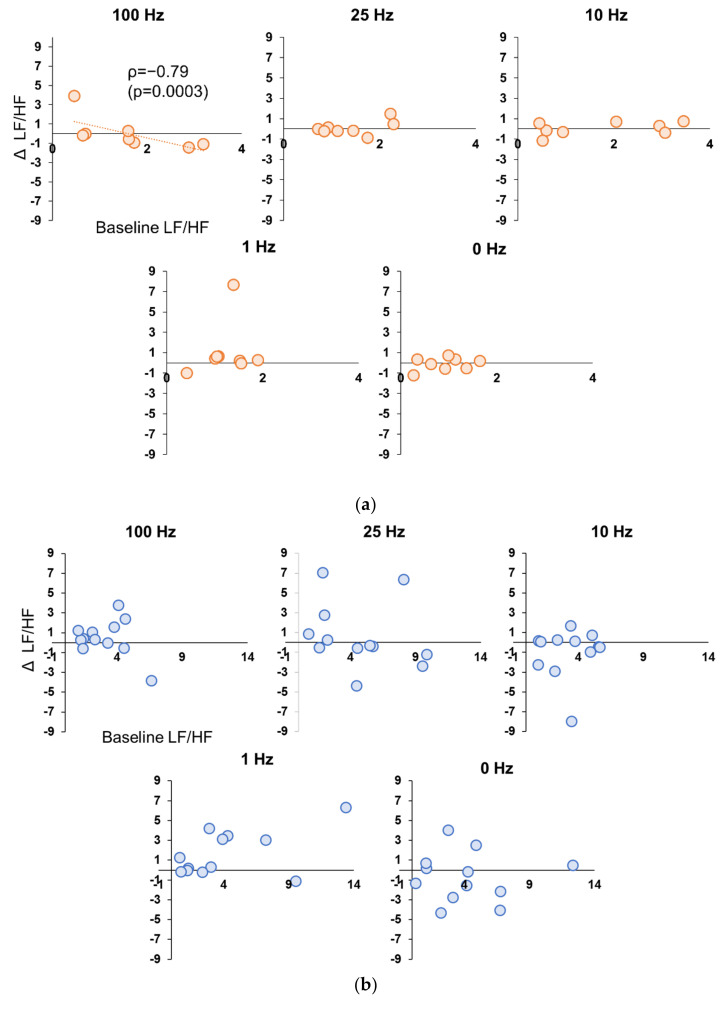
(**a**) Correlation between Baseline LF/HF value and decrement during stimulation period in female participants in Experiment 1. A significant negative correlation was found only for the 100 Hz condition (*p* = 0.0003). (**b**) Correlation between Baseline LF/HF value and decrement during stimulation period in male participants in Experiment 1. No significant correlation was found in any of the conditions.

**Figure 6:**
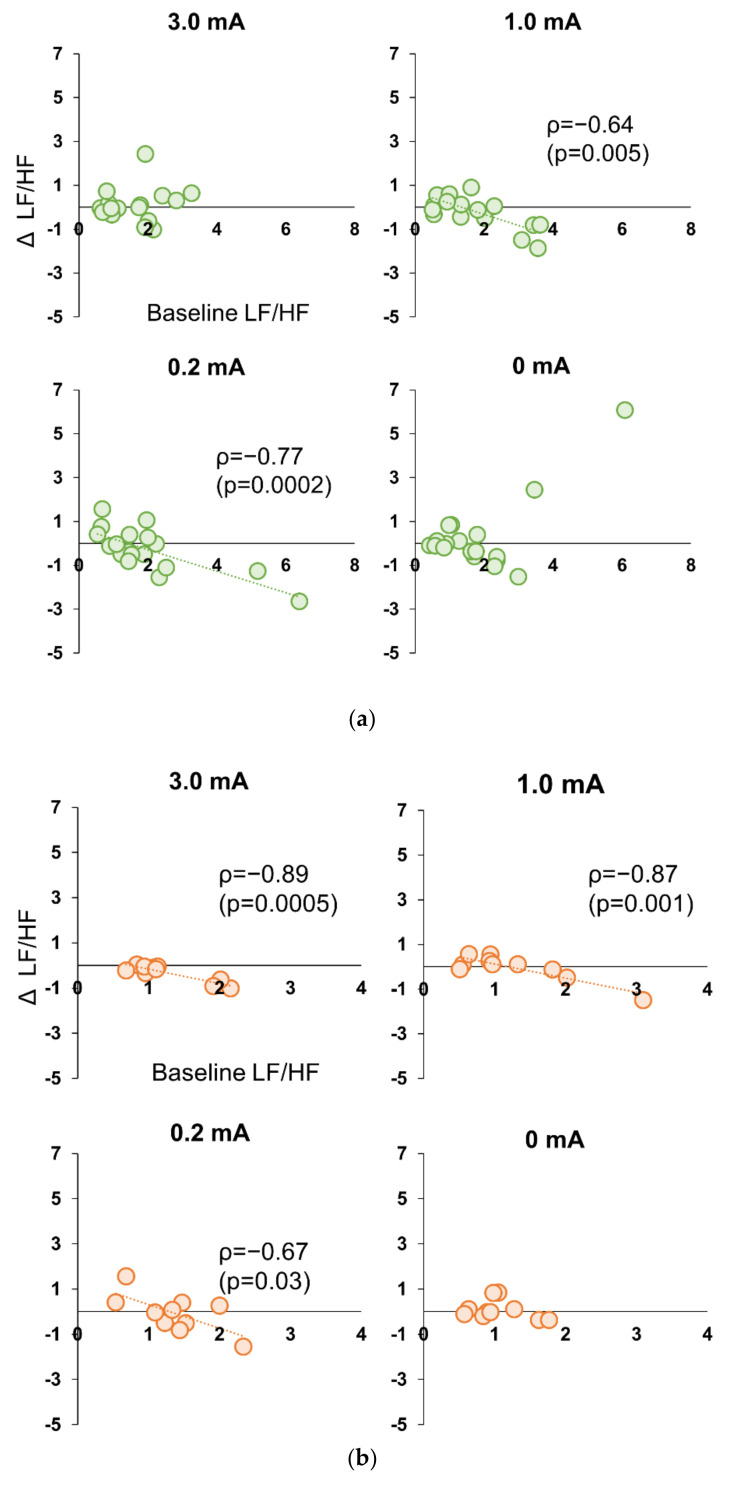

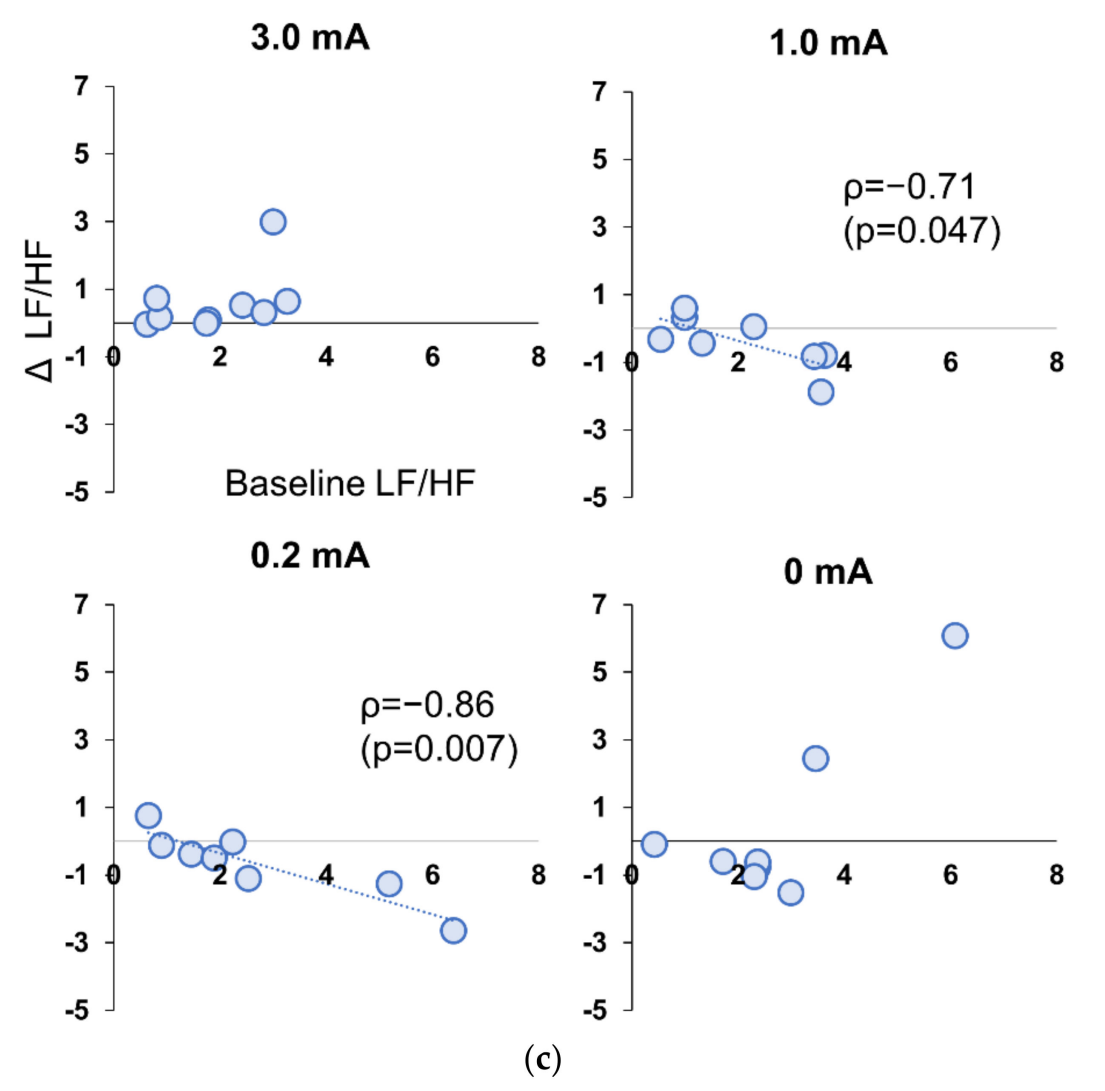
(**a**) Correlation between Baseline LF/HF value and decrement during stimulation period in all participants in Experiment 2. Significant negative correlations were found in the 0.2 mA and 1.0 mA conditions (*p* < 0.005). (**b**) Correlation between Baseline LF/HF value and decrement during stimulation period in female participants in Experiment 2. Significant negative correlations were found for all conditions except 0 mA (*p* < 0.001). (**c**) Correlation between Baseline LF/HF value and decrement during stimulation period in male participants in Experiment 2. Significant negative correlations were found in the 0.2 mA and 1.0 mA conditions (*p* < 0.05).

In Experiment 1, there were no significant correlations between the values at baseline and change in the values in stimulation period for HR, RMSSD, and LF/HF, for all participants in either condition. However, when only female participants were selected, there was a significant strong negative correlation (ρ = −0.881, *p* = 0.004) between the Baseline values for LF/HF only in the 100 Hz condition and the amount of change during stimulation (Figure 5a,b). In Experiment 2, there was a significant negative correlation between Baseline LF/HF values and change during stimulation only in the 1.0 mA (ρ = −0.636, *p* = 0.005) and 0.2 mA conditions (ρ = 0.768, *p* = 0.0002) for all subjects (Figure 6a). In Experiment 2, when sex differences were examined, significant negative correlations were also found in 3.0 mA (ρ = −0.894, *p* = 0.0005), 1.0 mA (ρ = −0.868, *p* = 0.001), and 0.2 mA conditions (ρ = −0.894, *p* = 0.0005) for females (Figure 6b), whereas no correlation was found for 3.0 mA in male subjects, and significant negative correlations were found only at 1.0 mA (ρ = −0.714, *p* = 0.005) and 0.2 mA (ρ = −0.857, *p* = 0.007) (Figure 6c).

### 3.6. Numerical Rating Scales: NRS in Subjective Discomfort Among Stimulus Conditions in Experiment 1

Subjective ratings of discomfort during tVNS stimulation at 100 Hz in Experiment 1 were significantly higher (*p* < 0.01) than for all other frequency conditions, with an NRS mean and standard error of 4.40 ± 0.36. The NRSs for the 25 Hz, 10 Hz, and 1 Hz conditions were 3.45 ± 0.32, 3.01 ± 0.34, and 2.36 ± .028, respectively, indicating significantly higher discomfort compared to the 0 Hz condition (*p* < 0.01); however, there were no significant differences in experienced discomfort between these frequencies (*p* > 0.05).

### 3.7. Perceptual Threshold and NRS in Subjective Discomfort among Stimulus Conditions in Experiment 2

In Experiment 2, the mean (±standard error) sensory threshold for the participants was 0.34 (±0.01 mA). The NRS for the 3.0 mA condition was significantly higher than all other current intensities [5.39 ± 0.23 (*p* < 0.01)]. The NRS for the 1.0 mA condition was 2.56 ± 0.28, significantly higher than the 0.2 mA (below sensory threshold) and 0 mA conditions (*p* < 0.01).

### 3.8. Adverse Events

In both Experiments 1 and 2, no subjects showed excessive HR reduction, headache, pain, and/or redness at the stimulation site, during or after tVNS stimulation.

## 4. Discussion

The purpose of this study was to determine the effects of various stimulus frequencies and current intensity parameters on autonomic nervous system activity, induced by 1 min continuous tVNS to the left cymba concha in healthy participants. Understanding these effects in healthy conditions provides the framework for determining a reliable tVNS protocol. We found that tVNS significantly reduced HR when delivered with a stimulus frequency of 100 Hz, a stimulus current intensity of 3.0 mA. Furthermore, HR was more effectively reduced at the high-frequency band of 100 Hz with a stimulus intensity of 3.0 mA. Interestingly, in the condition, LF/HF decreased during stimulation only in female subjects with higher baseline LF/HF. On the other hand, although no decrease in HR was observed at 1.0 mA and 0.2 mA current intensities, a negative correlation between baseline and LF/HF during stimulation was observed in all subjects, including males, suggesting the existence of sex differences in tVNS stimulation intensity suitable for parasympathetic nerve activity.

Experiment 1 revealed that stimulation in the high-frequency band of 100 Hz was more effective in inputting vagal afferent fibers, suggesting that different frequencies may yield different effects on HR. In epilepsy model rats, high-frequency tVNS (above 80 Hz) increased the frequency of neuronal firing in the LC and significantly suppressed seizures [21,29]. In human participants, tVNS delivered at 100 Hz to the left cymba concha induced significantly higher activity in brainstem nuclei, such as the NTS and LC, than low-frequency stimulation at 25 Hz or lower; furthermore, the activity of the LC correlated with the amount of increase in HF, a measure of parasympathetic activity in HRV [20]. This suggests that the strength of signal transmission by the afferent fibers of the vagus nerve increases in a frequency-dependent manner. In this study, the input to the NTS through vagus afferents demonstrated the greatest increase at 100 Hz tVNS, which may have produced an effective suppression of HR.

The mechanism for the HR suppression via tVNS is suggested to be the activation of excitatory inputs to the caudal ventrolateral medulla medulla by afferent fibers via the NTS; this would consequently suppress the rostral ventrolateral medulla, the source of excitatory drive of the sympathetic nervous system [30]. Previous studies indicate that tVNS activates a wide range of brain regions involved in the autonomic nervous system control [13,14,31] and that the heart-evoked potential, which reflects the cardiac interoceptive input, increases in these areas during tVNS. The involvement of higher-order autonomic control in addition to the reflexive heart rate inhibition at the medullary level may explain this phenomenon [32]. Therefore, tVNS could suppress HR at the level of the medulla and the upper centers involved in autonomic control in the present experiment.

Analysis of the 5 s time course of HR highlighted a decrease in HR immediately after stimulus onset, which reached its lowest value in 10 s in all frequency conditions. However, the time required to return to baseline value differed among the conditions, with the 100 Hz condition requiring the longest time. Therefore, the longer duration of effectiveness in tVNS likely resulted in a more pronounced reduction in HR, suggesting that stimulus duration is also an important factor that should be considered in determining a reliable and effective tVNS protocol. Consistent with previous studies, a HR rebound was observed immediately after termination of the stimulation in all conditions, including the control condition [15]. However, Badran et al. stimulated the earlobe under sham conditions, whereas the present study used 0 Hz condition as a control condition and did not apply an electric current. A rebound was observed even at 0 Hz in this experiment, making it unlikely that the effect was caused by tVNS. Considering the influence of verbal communication for NRS acquisition, we recorded an NRS rating during the rest period after the end of Post 2 in Experiment 2 to examine closely whether the rebound was caused by tVNS.

A decrease in HR was only observed with stimulation at 3.0 mA and 100 Hz, but not at 1.0 mA and 0.2 mA, suggesting that a certain level of current intensity is required to effectively decrease the HR. In the 5 s time series analysis, HR decreased significantly from 35 s after the onset of stimulation to 10 s after the end of stimulation in the 3.0 mA condition compared to baseline, while in the 1.0 mA condition, HR decreased significantly at 50 s after the onset of stimulation compared to baseline. The 0.2 mA condition did not show a significant decrease in HR. The post-stimulus HR rebound observed in Experiment 1 was not observed immediately after the termination of stimulation in all conditions, but a large rebound was observed when the NRS was taken during the resting period after Post 2 in Experiment 2. Therefore, the HR rebound after the termination of stimulation in Experiment 1 was not due to homeostatic plasticity, but rather to the physiological reaction to the verbal NRS response. In animal studies on the application of tVNS for cardiac disease, high-intensity stimulation to vagal efferent fibers induces atrial fibrillation and low-intensity VNS has been shown to suppress atrial fibrillation [7,10,24,30,33,34,35,36]. However, atrial fibrillation cannot be induced unless the HR is reduced by 40% or more [36]. Therefore, tVNS to the left cymba concha at 3.0 mA effectively reduced the HR within a safe range in vivo. On the other hand, it has been reported that the LC is a very-low-threshold nucleus and the LC activity is thought to be inhibited by the nearby trigeminal nucleus when stimulated at higher intensities [37]. In addition, the simultaneous application of a certain rehabilitation approach and moderate tVNS is said to facilitate neural plasticity at the associated region of the cortex [38]. Although tVNS is reported to yield variable results, this study suggests that there is a quantifiable stimulus intensity that induces plasticity, so it is important to set the intensity according to the purpose in order to maximize the effect of tVNS.

The possibility that there is an appropriate stimulus intensity for the plastic change we want to induce was supported in the LF/HF in our experiment. A significant negative correlation was found between Baseline LF/HF value and change during stimulation for all participants in Experiment 2 only at 1.0 mA and 0.2 mA intensity, but no correlation was found at 3.0 mA. In a previous study, tVNS to the left tragus for 5 to 15 min increased parasympathetic activity after stimulation in participants with higher sympathetic activity before stimulation [26,39]. The high-frequency, low-intensity tVNS to the left cymba concha for 1 min used in this experiment was also effective in activating parasympathetic activity in participants with high sympathetic baseline activity. More interestingly, the LF/HF ratio at baseline and the change during stimulation were influenced by sex. The strongest negative correlation was found at 3.0 mA for female participants compared to the other stimulus intensities in Experiment 2 and was consistent in Experiment 1. Regarding the effect of sex differences on parasympathetic activity induced by tVNS, Couck et al. reported that standard deviation of the RR intervals (SDNN), a measure of parasympathetic activity, was greatly enhanced in female compared to male subjects after tVNS [16]. Although, no sex differences in the morphological characteristics of vagus nerve fibers were reported in an animal study [40], functional differences in the vagus nerve between sexes are reported that information from organs, such as the uterus and ovaries, is transmitted to the brainstem via vagal afferents, and that estrogen receptors are present in neurons in the vagus ganglion [41]. In addition, since autonomic nervous system activity is also associated with disorders specific to women, such as premenstrual syndrome and dysmenorrhea [42,43], functional sex differences in the transmission of information by vagal afferents may exist. Therefore, these structural and functional sex differences in vagal afferents may have influenced the effects of the tVNS on autonomic activity in this study.

There are two major limitations to this study. First, the experiment was conducted using a combination of stimulus frequency and current intensity parameters, while the pulse width was kept constant at 250 µs. Since it has been reported that tVNS pulse width also affects HR [15], different pulse widths may cause different outcomes. Therefore, the results of this experiment cannot be regarded as an optimal stimulation method, but only as a demonstration of the effects of tVNS stimulation frequency and current intensity on HR and HRV. Another major limitation in this study is that although sex differences were observed in the effects of tVNS on HRV in this experiment, the female hormones estrogen and progesterone are known to affect neuronal plasticity in the brain [44,45]. Thus, the results may be influenced by hormonal changes in the menstrual cycle or when using hormone-based contraceptives. This experiment did not consider the effect, so more detailed studies are needed in the future.

## 5. Conclusions

Our findings indicate that tVNS to the left cymba concha can effectively reduce HR when applied with a stimulation frequency of 100 Hz, a stimulation current intensity of 3.0 mA, and over 250 µs, to induce autonomic nervous system activity. Furthermore, higher baseline sympathetic activity and sex differences may yield a greater parasympathetic response to tVNS and the modulation of HRV. The results of the present experiments suggest that the stimulus parameters and sex difference should be taken into account and recommend the optimal dose in the clinical application of tVNS, such as atrial fibrillation, epilepsy, and stroke, to ensure that patients receive maximum possible benefit from the treatment.

## Data Availability

The datasets presented in this article are not readily available because the datasets involve unfinished research projects. If necessary, requests to access the datasets should be directed to the corresponding author.
